# Flagellin *O*-linked glycans are required for the interactions between *Campylobacter jejuni* and *Acanthamoebae castellanii*

**DOI:** 10.1099/mic.0.001386

**Published:** 2023-08-23

**Authors:** Fauzy Nasher, Brendan W. Wren

**Affiliations:** ^1^​ London School of Hygiene and Tropical Medicine, London, UK

**Keywords:** *Acanthamoebae*, *Campylobacter jejuni*, flagella, glycosylation, MAMPs, PRRs

## Abstract

The predation and engulfment of bacteria by *Acanthamoebae* facilitates intimate interactions between host and prey. This process plays an important and underestimated role in the physiology, ecology and evolution of pathogenic bacteria. *Acanthamoebae* species can be reservoirs for many important human pathogens including *Campylobacter jejuni. C. jejuni* is the leading cause of bacterial foodborne enteritis worldwide, despite being a microaerophile that is incapable of withstanding atmospheric levels of oxygen long-term. The persistence and transmission of this major pathogen in the natural environment outside its avian and mammalian hosts is not fully understood. Recent evidence has provided insight into the relationship of *C. jejuni* and *Acanthamoebae* spp. where *Acanthamoebae* are a transient host for this pathogen. Mutations to the flagella components were shown to affect *C. jejuni–A. castellanii* interactions. Here, we show that the motility function of flagella is not a prerequisite for *C. jejuni–A. castellanii* interactions and that specific *O*-linked glycan modifications of the *C. jejuni* major flagellin, FlaA, are important for the recognition, interaction and phagocytosis by *A. castellanii*. Substitution of the *O*-linked glycosylated serine 415 and threonine 477 with alanine within FlaA abolished *C. jejuni* interactions with *A. castellanii* and these mutants were indistinguishable from a Δ*flaA* mutant. By contrast, mutation to serine 405 did not affect *C. jejuni* 11168H and *A. castellanii* interactions. Given the abundance of flagella glycosylation among clinically important pathogens, our observations may have a wider implication for understanding host–pathogen interactions.

## Full-Text

## Data availability

All data generated or analysed during this study are included in the manuscript and supporting files.

## Introduction

Bacteria and free-living amoebae (FLA) interactions were described as a predator–prey relationship, consequently regulating bacteria population [1]. However, over the last few decades some bacteria and amoebae interactions were reported to be mutually beneficial [2]. The limited capabilities of non-spore-forming bacteria to withstand hostile environments may have led to the evolutionary need to interact with a robust host [3]. This co-evolutionary evidence has been observed in *Legionella pneumophila,* and its intra-amoebal survival influences its virulence and pathogenicity [4]. More recently, this has also been observed in *Campylobacter jejuni* [5].

Some bacteria mediate uptake by amoebae using secretory organelles [6], whilst uptake of others is predominantly driven by pattern recognition receptors (PRRs) that simultaneously bind microbe-associated molecular patterns (MAMPs) [6]. The same relationships are observed with phagocytes from warm-blooded mammals [7]. The *Acanthamoeba* genome has multiple putative PRRs that have predicted opsonophagocytic orthologues in higher organisms [8].

*C. jejuni* is the leading cause of foodborne gastroenteritis, with infection thought to surpass those of *Salmonella enteritis* [9]. The microaerophilic nature of *C. jejuni* and the inability to grow in the natural environment outside of its warm-blooded hosts, yet is the most prevalent bacterial enteropathogen, remains puzzling. *Acanthamoebae* was hypothesized to provide *C. jejuni* refuge [10], and indeed, we showed that *Acanthamoebae* spp. are a transient host for *C. jejuni *, and the bacterium is capable of subverting *Acanthamoeba* killing by employing similar survival strategies as it does in the cells of warm-blooded hosts [5, 11].

Previously, we reported the inability of *A. castellanii* to phagocytose *C. jejuni* flagella mutants [11]. Here, we show that properties unrelated to motility function of flagella are crucial for *A. castellanii* interactions, and that the *O*-linked glycosylation modification of the flagellin facilitates recognition, capture and phagocytosis. The evolution of *C. jejuni* towards a major pathogen may have been shaped by frequent encounters with free-living amoebae.

## Methods

### Bacteria and amoebae cultures

Bacteria were stored using Protect bacterial preservers (Technical Service Consultants) at − 80 °C. *C. jejuni* strains were streaked on blood agar (CBA) plates containing Columbia agar base (Oxoid) supplemented with 7% (v/v) horse blood (TCS Microbiology) and Campylobacter Selective Supplement (Oxoid) with or without selective antibiotics, and grown at 37 °C in a microaerobic chamber (Don Whitley Scientific), containing 85% N_2_, 10% CO_2_ and 5% O_2_ for 48 h. Bacteria were grown for a further 16 h at 37°C prior to use.

*Acanthamoeba castellanii* CCAP 1501/10 (Culture collection of Algae and protozoa) was cultured to confluence at 25°C in peptone yeast and glucose (PYG) [11]. Viability was determined using light microscopy.

*Dictyostelium discoideum* Ax2(Ka) wild-type cells were grown in axenic conditions at 22 °C in HL5c medium (Formedium) including glucose supplemented with vitamins and micro-elements, as described previously [12].

### *C. jejuni* mutant transformation

*C. jejuni* 11168H and its Δf*laA* and Δ*flaB* mutants were previously described [11]. Site-directed mutagenesis was performed using NEBuilder HiFi DNA Assembly following the manufacturer’s protocol (New England Biolabs). Briefly, *C. jejuni* 11168H in which most of the *flaA* gene was replaced with a Kan^r^ cassette to generate ∆*flaA* was used to generate ∆*flaA+flaA*_S406A_, ∆*flaA*+f*laA*_S415A_ and ∆*flaA+flaAT_T477A_*. The wild-type *flaA* gene with intact native promoter was amplified using two sets of primers (Table S1, available with the online version of this article) with overlapping sequence that introduced the desired mutation. The fragment was inserted into plasmid pRRA [apramycin-resistant (Ap^r^)]; this plasmid introduces genes into the conserved multi-copy rRNA gene clusters on the chromosome [13]. Plasmids with desired site-directed mutation were cloned in *Escherichia coli* DH5α, and transformants were selected on LB agar supplemented with apramycin and ampicillin. The resultant plasmids were electroporated into *C. jejuni* with selection for Ap^r^ and Kan^r^ as previously described [14]. ∆*flaA+flaA*_S406A_, ∆*flaA*+f*laA*_S415A_ and ∆*flaA+flaAT_T477A_* were screened for motility and autoagglutination, motility was restored in all strains and ∆*flaA*+*flaA*_S406A_ was not defected with autoagglutination, as described previously [15].

### *C. jejuni* invasion and survival assay

Invasion and survival assays were performed as previously described [11]. Briefly, *C. jejuni* 11168H and its mutants were incubated with a monolayer of 10^6^ *A. castellanii* at an m.o.i. of 200 for 3 h at 25 °C in 2 ml PYG media. The monolayer was washed three times with PBS and incubated for 1 h in PYG media containing 100 µg ml^−1^ gentamicin. Bacterial cells were harvested by lysing amoebae in distilled water containing 0.1 % (v/v) Triton X-100 for 10 min at room temperature. The suspension was centrifuged for 10 min at 4000 *g*, and the resultant pellet was resuspended in 1 ml PBS and enumerated for colony-forming units.

### Live-cell imaging

Wild-type *C. jejuni* and its Δ*flaA*, Δf*laB*, ∆*flaA+flaA*_S406A_, ∆*flaA*+f*laA*_S415A_ and ∆*flaA+flaAT_T477A_* mutants were stained with SYTO 9 Green Fluorescent following the manufacturer’s protocol (Thermofischer) before live-cell imaging; 10^6^ of adherent *A. castellanii* in PYG media were infected with bacteria at an m.o.i. of 200 in 35 mm μ-Dish devices (IBIDI). Interactions were monitored in the green region (excitation and emission wavelengths of 488/510 nm). Dead bacteria were stained with propidium iodide and monitored in the red region (excitation and emission wavelengths of 550/720 nm). Confocal laser scanning micrographs were obtained using an inverted Zeiss LSM 880 confocal microscope (Zeiss). Images were taken at 5 s intervals for time lapse experiments.

## Results and discussion

### Immobile *C. jejuni* do not interact with *A. castellanii*

**Fig. 1.**
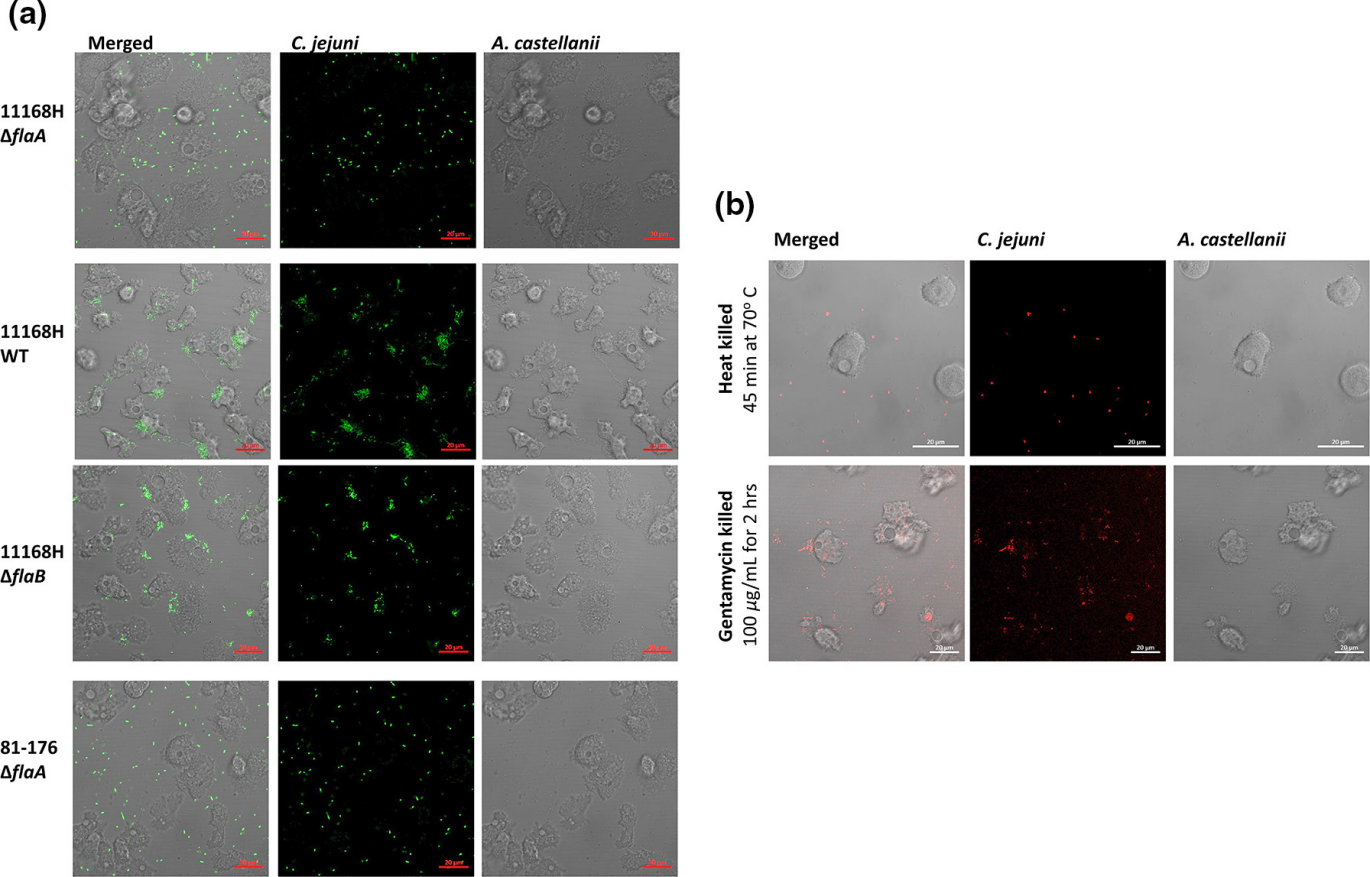
*
C. jejuni* strain 11168H and ∆*flaA*, ∆*flaB* mutants and strain 81-176 and its ∆*fl*aA mutant interaction with *A. castellanii*. (**a**) *C. jejuni* cells from a 16 h CBA plate were suspended in PYG media and stained with SYTO 9 Green Fluorescent for 30 min, OD_600nm_ was adjusted to 0.1 (this is equivalent to ∼10^8^; m.o.i of 200), and interactions were monitored by time-lapse imaging for 5 h. (**b**) Heat-killed (70 °C for 45 min) or gentamycin-killed (100 µgml^–1^ for 2 h) bacterial cells were stained with a Live/Dead Bactlight bacteria viability staining kit for 30 min, and bacteria at an m.o.i. of 200 were co-incubated with *A. castellanii*. Images are representative of 2 h time-lapse experiments of at least three biological replicates. Images were obtained using a confocal microscope with ×63 oil objective; bar=20 µm. See also Video files SV1–SV6.

*C. jejuni* requires its flagella for gut colonization, motility, secretion of virulence factors [16] and autoagglutination. Flagella promotes interactions with host cells, and biofilm and micro-colony formation [17]. *C. jejuni* 11168H flagella mutants abolished uptake by *A. castellanii* [11]. Here, live-cell imaging revealed that the *C. jejuni* 11168H ∆*flaA* (major flagellin) mutant did not interact with *A. castellanii* (Video SV1), and this phenotype was also apparent for the *C. jejuni* strain 81-176 ∆*flaA* mutant (Video SV2). The *C. jejuni* ∆*flaB* (minor flagellin) mutant showed interactions with *A. castellanii*, but phagocytosis was reduced and delayed (Video SV3) relative to the parental strain (Video SV4) (Fig. 1a).

Given that *C. jejuni* ∆*flaA* mutants are immobile, and motility was speculated to be important for bacterial encounters with *Acanthamoebae* [18], we tested whether this defect hindered interactions with *A. castellanii*. Heat- (70 °C for 45 min) or gentamycin- (100 µgml^–1^ for 2 h) killed bacteria were used to ablate the motility properties of the flagella. Gentamycin-killed bacteria interacted with *A. castellanii* (Video SV5), as evident by bacteria-filled vacuoles, contrary to heat-killed *C. jejuni* (Video SV6) (Fig. 1b). Killed bacteria were non-motile, and therefore it was likely that antibiotic-killed bacteria maintained intact cell surface proteins, including the flagella. This implied that properties unrelated to motility are crucial during *C. jejuni–A. castellanii* interactions.

*C. jejuni* aggregate on the surface of *Acanthamoebae* prior to phagocytosis [10, 11], although it is now apparent that this aggregation is not essential to induce a productive internalization given that phagocytosis of the ∆*flaB* mutant was dramatically reduced. Interestingly, this aggregation phenomenon was also observed during *Listeria monocytogenes* interactions with *Acanthamoeba* and was termed ‘backpacking’ [18]. This phenotype was concluded to be driven by amoebae locomotion [18]. By contrast, *C. jejuni* aggregation was not a consequence of amoebae movement, and considering many other bacterial species that interact with *Acanthamoebae* are flagellated and their interactions are intentional [19, 20], it is likely, at least for *C. jejuni *, that an intact flagella facilitates adhesion on the surface of the amoebae, which in turn triggers a productive internalization. In fact, *O*-linked glycosylation of flagellae of the bacterial pathogens *Helicobacter pylori* [21] and *Aeromonas hydrophila* [22] have been shown to promote their interactions with epithelial cells.

### *O*-linked glycosylation of FlaA is required for *C. jejuni–A. castellanii interactions*

*Campylobacter* spp. FlaA is post-translationally modified (PTM) by *O*-linked glycosylation which adds pseudaminic acid, and in some strains, additionally legionaminic acid [23, 24]. FlaA was shown to be glycosylated at 19 serine and threonine sites [15]. Mutations to specific serine or threonine sites in FlaA showed autoagglutination defects, whilst some mutations showed defects in motility due flagella destabilization in *C. jejuni* strain 81-176 FlaA [15]. These glycosylated sites are highly conserved among *C. jejuni* strains [24].

We substituted serine 406, 415 and threonine 477 with alanine in *C. jejuni* strain 11168H. These corresponded to S408, S418 and T481 in strain 81-176, respectively [15]. S415 and T477 were chosen because they retained motility but had the largest defects in autoagglutination; this attribute is associated with adhesion, hydrophobicity, biofilm formation and microcolony formation, whereas mutation to S406 was chosen due to lack of a distinct phenotype compared to the parental strain [15].

**Fig. 2.**
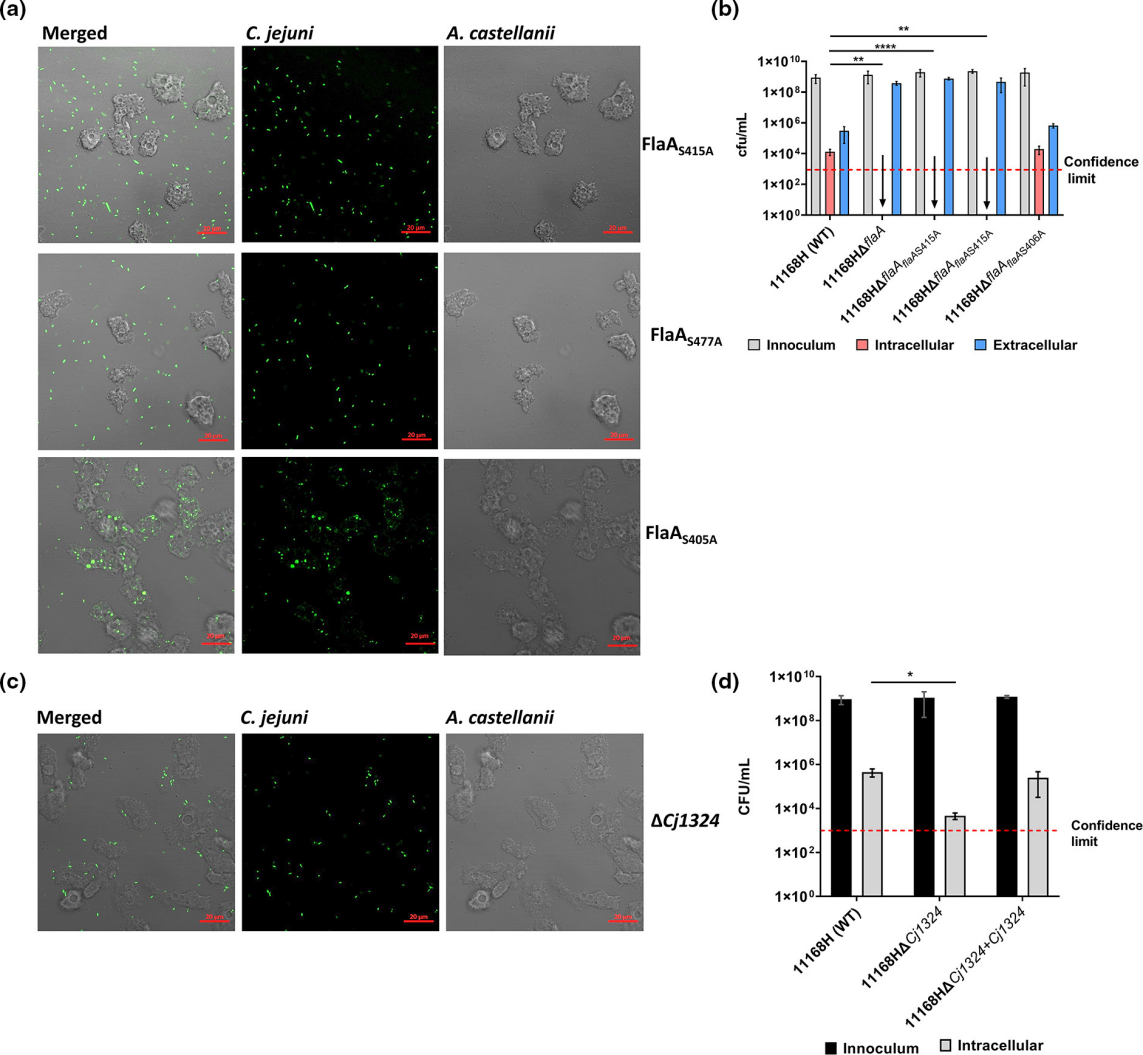
Mutation to specific amino acids within FlaA abolishes *C. jejuni* interactions with *A. castellanii*. (**a**) *C. jejuni* 11168H ∆*flaA+flaA*_S406A_, ∆*flaA+flaA*_S415A_ and ∆*flaA*+flaA_T477A_ mutant interactions with *A. castellanii* and (b) enumeration of live phagocytosed bacteria after 4 h of co-incubation. (c) Live-cell imaging of *C. jejuni* 11168H Δ*Cj1324* interactions with *A. castellanii*, and (d) intracellular recovered wild-type *C. jejuni* 11168H, Δ*Cj1324* and Δ*Cj1324+Cj1324* bacteria. Live-cell imaging was performed after staining the cells with SYTO 9 Green Fluorescent for 30 min, with OD_600nm_ adjusted to 0.1 (equivalent to ∼10^8^; m.o.i.=200). Images were captured with an inverted confocal microscope with ×63 oil objective; bar=20 µm. Results are representative of 3 h time-lapse experiments of at least three biological replicates performed independently. Enumeration of live phagocytosed bacteria after 4 h co-incubation (including 1 h gentamycin treatment) with *A. castellanii*; data are presented as c.f.u. ml^–1^ of three biological replicates performed on independent days; data analysis was performed in GraphPad Software using a two-way ANOVA, and data are presented as standard deviation (sd). Red dashed line represents confidence limit. *P<0.05; **P<0.002; ****P<0.0001. See also Video files SV7–SV10.

S415 and T477 substitutions with alanine abolished *C. jejuni–A. castellanii* interactions and phagocytosis (Fig. 2a; Videos SV7 and SV8, respectively). This phenotype was indistinguishable from the ∆*flaA* mutant. Interestingly, substitution of S406 with alanine did not affect *C. jejuni–A. castellanii* interactions (Fig. 2a; Video SV9). Enumeration of intracellular bacteria was in line with live-cell imaging; we did not recover intracellular *C. jejuni* ∆*flaA+flaA*_S415A_ and ∆*flaA*_T477A_, similar to the ∆*flaA* mutant, but *C. jejuni* ∆*flaA+flaA*_S406A_ were recovered similarly to the parental strain (Fig. 2b). The full extent of *C. jejuni* flagellin glycosylation remains to be fully explored, and therefore we do not rule out that other glycosylated sites might result in further phenotypes.

Unlike most *C. jejuni* strains, 11168H flagellin is modified with both pseudaminic acid and legionaminic acid and derivatives [24]. Characterization of the *O*-linked glycosylation island revealed mutation to *Cj1324*, which is important for legionaminic acid biosynthesis on *C. jejuni* 11 168 flagellin. Although motile, the Δ*Cj1324* mutant showed defects in hydrophobicity, autoagglutination and chicken colonization. Structural analyses of Δ*Cj1324* flagella revealed the absence of two legionaminic acid glycan modifications [25]. Here, the Δ*Cj1324* mutant showed reduced interactions, but was phagocytosed by *A. castellanii* (Fig. 2c; Video SV10). Intracellular bacteria were significantly (*P*<0.05) reduced relative to the wild-type strain (Fig. 2d). This intracellular reduction was in line with the reduced interaction. This observation implies that recognition and uptake of *C. jejuni* by *A. castellanii* may be dependent on the more conserved pseudaminic acid and derivative glycoform modifications.

The ubiquitous nature of *Acanthamoebae* may suggest a shared ecological niche with *C. jejuni *. Frequent interactions may have been a ‘melting pot’ for *C. jejuni* evolution. Considering flagella phase variability [26], our findings suggest that *C. jejuni* phase variation may have been an adaptive response to FLA. Indeed, we show that with co-incubation of *C. jejuni* with the soil amoeba, *Dictyostelium discoideum*, *C. jejuni* and *D. discoideum* interactions were randomly driven and that upon phagocytosis, *C. jejuni* were digested within minutes (Fig. S1a, b and Video SV11).

Flagellum glycosylation is considered a MAMP and binds to PRRs on a variety of phagocytes which trigger their uptake, including phagocytes in warm-blooded mammals [21, 27]. Given the abundance of flagella PTM in nature, our novel findings may have wider implications for other bacterial species such as *Burkholderia* spp., *Listeria* spp. and *Legionella* spp.*,* in which *Acanthamoebae* play important role in their life cycles [6]. However, it is also worth noting that whilst some bacteria utilize a single monosaccharide in their flagella [21], *C. jejuni* flagellin is one of the most heavily glycosylated proteins identified to date [28]. Therefore, the interactions observed here may be different and more evolved compared to other bacteria species.

In conclusion, studying *C. jejuni */*Acanthamoebae* interactions represents a useful model to understand how this enigmatic pathogen has evolved to be the leading cause of foodborne enteritis.

## Supplementary Data

Supplementary material 1

Supplementary material 2
