# Supplementary material for: Flagellin O-linked glycans are required for the interactions between Campylobacter jejuni and Acanthamoebae castellanii
Source: Microbiology (Reading). 2023 Aug 23;169(8):001386. doi: 10.1099/mic.0.001386 (PMC10482376; doi:10.1099/mic.0.001386)
Supplement: Supplementary material 1 [file mic-169-1386-s001.pdf]

Flagellin *O*-linked glycans are required for the interactions between *Campylobacter jejuni* and *Acanthamoebae castellanii*

Fauzy Nasher[1]*, Brendan W. Wren[1]*

[1]London School of Hygiene and Tropical Medicine, London, United Kingdom

*Address correspondence to **Fauzy Nasher**: fauzy.nasher1@lshtm.ac.uk (ORCiD) and **Brendan W. Wren**: brendan.wren@lshtm.ac.uk (ORCiD)

Supplementary material

Table S1

**Mutation**	**Primer sets**
Serine 406	**Universal F:** ACACTCTAGATATTTTTTTGATTGCACGATATAG<br>**S405R1:** ATCCTGCGCTACTCATATAGGCACTAACAG<br>**S405F2:** CTATATGAGTAGCGCAGGAGCAGGATTTTC<br>**Universal R:** ACACCAATTGGCTTTTGTAAACTACTGTAGTAATC
Serine 415	**Universal F:** ACACTCTAGATATTTTTTTGATTGCACGATATAG<br>**S415R1:** TGAACCTGAAGAAAATCCACTTCCTGCG<br>**S415F2:** GTGGTTTTCTTCAGGTTCAGGTTATGCGGTAGG<br>**Universal R:** ACACCAATTGGCTTTTGTAAACTACTGTAGTAATC
Threonine 477	**Universal F:** ACACTCTAGATATTTTTTTGATTGCACGATATAG<br>**R1:** GCAACACCTGCTGTTTCATCTTTTACTCCAAAAG<br>**F2:** GATGAAACAGCAGGTGTTGCCACACTTAAAG<br>**Universal R:** ACACCAATTGGCTTTTGTAAACTACTGTAGTAATC

F=Forward
R=Reverse
Universal=Forward and reverse primers spanning the whole flaA gene including native promoter

Figure S1

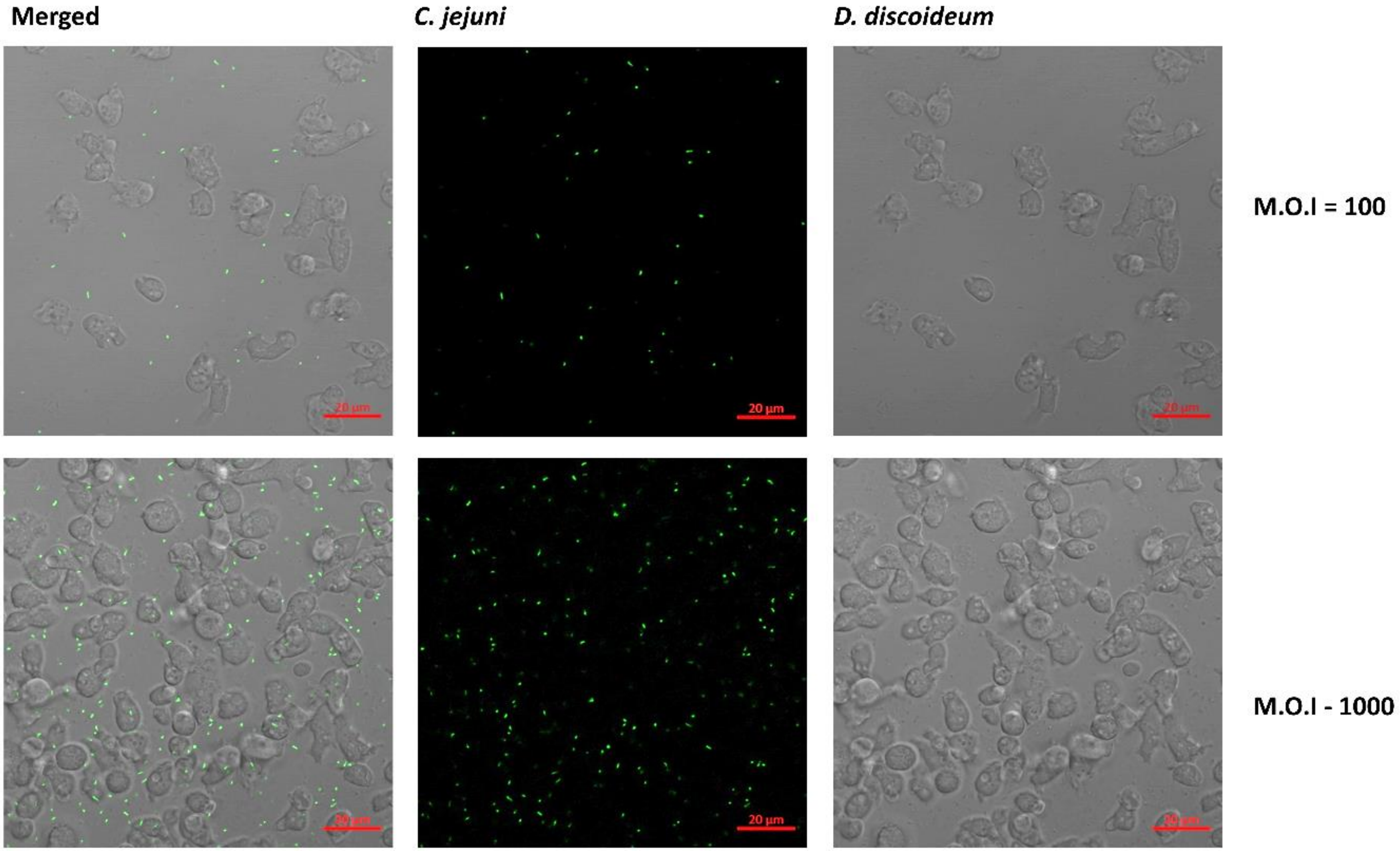

Figure S1a. ***C. jejuni*** **interactions with** ***D. discoideum*** **Ax2(Ka).** *C. jejuni* 11168H_{GFP} from a 16 hr CBA plate were suspended in HL5c medium OD_{600nm} was adjusted accordingly. *D. discoideum* Ax2(Ka) in HL5c media were infected with *C. jejuni* and interaction monitored by time-lapse imaging for 3 hr using inverted confocal microscope, Zeiss LSM-880 at x63 oil objective.

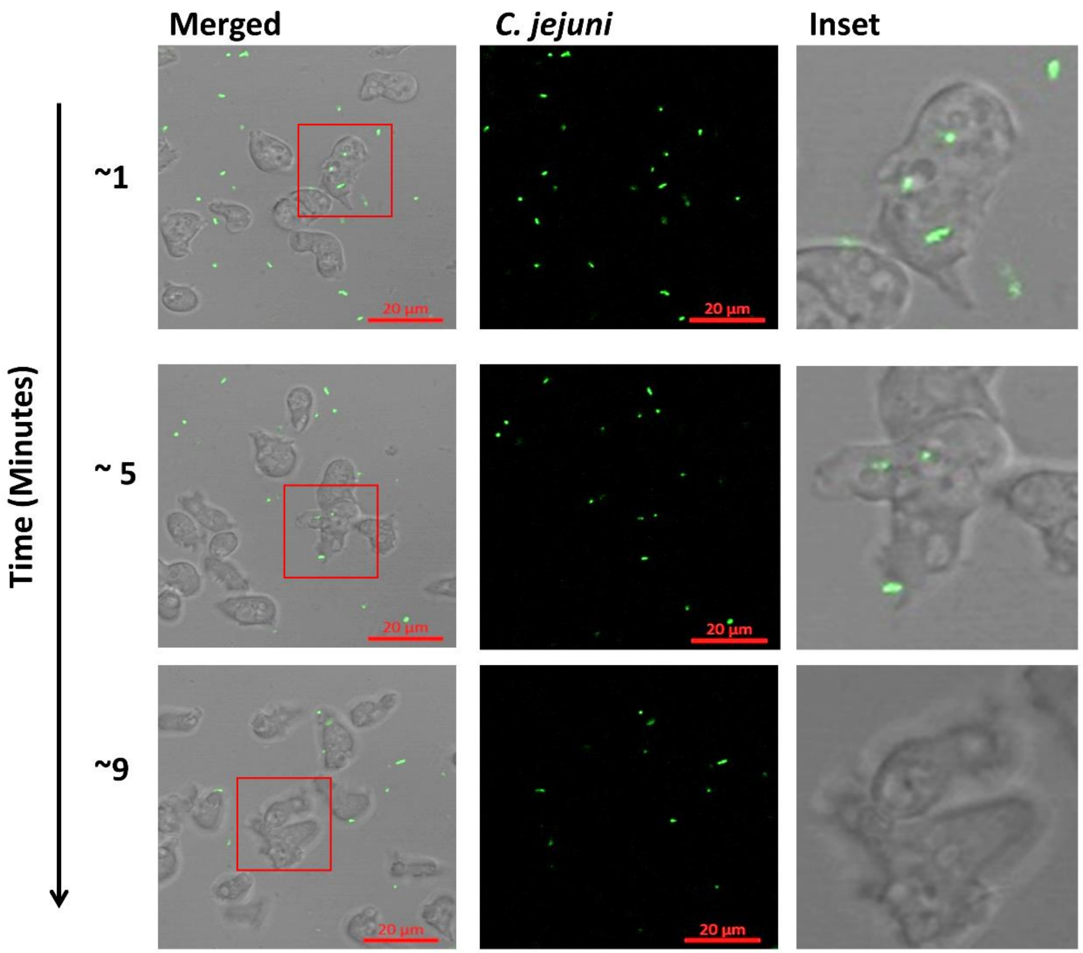

Figure S1b. *D. discoideum* Ax2(Ka) killing of intracellular *C. jejuni*. Time lapse images showing killing of *C. jejuni* 11168HGFP within minutes of internalization. *D. discoideum* Ax2(Ka) in HL5c media were infected with *C. jejuni* and interaction monitored by time-lapse using inverted confocal microscope, Zeiss LSM-880 at x63 oil objective (**Supplementary Video 11**).
