# Supplementary material for: Flagellin O-linked glycans are required for the interactions between Campylobacter jejuni and Acanthamoebae castellanii
Source: Microbiology (Reading). 2023 Aug 23;169(8):001386. doi: 10.1099/mic.0.001386 (PMC10482376; doi:10.1099/mic.0.001386)
Supplement: Supplementary material 2 [file mic-169-1386-s002.pptx]

## Slide 1
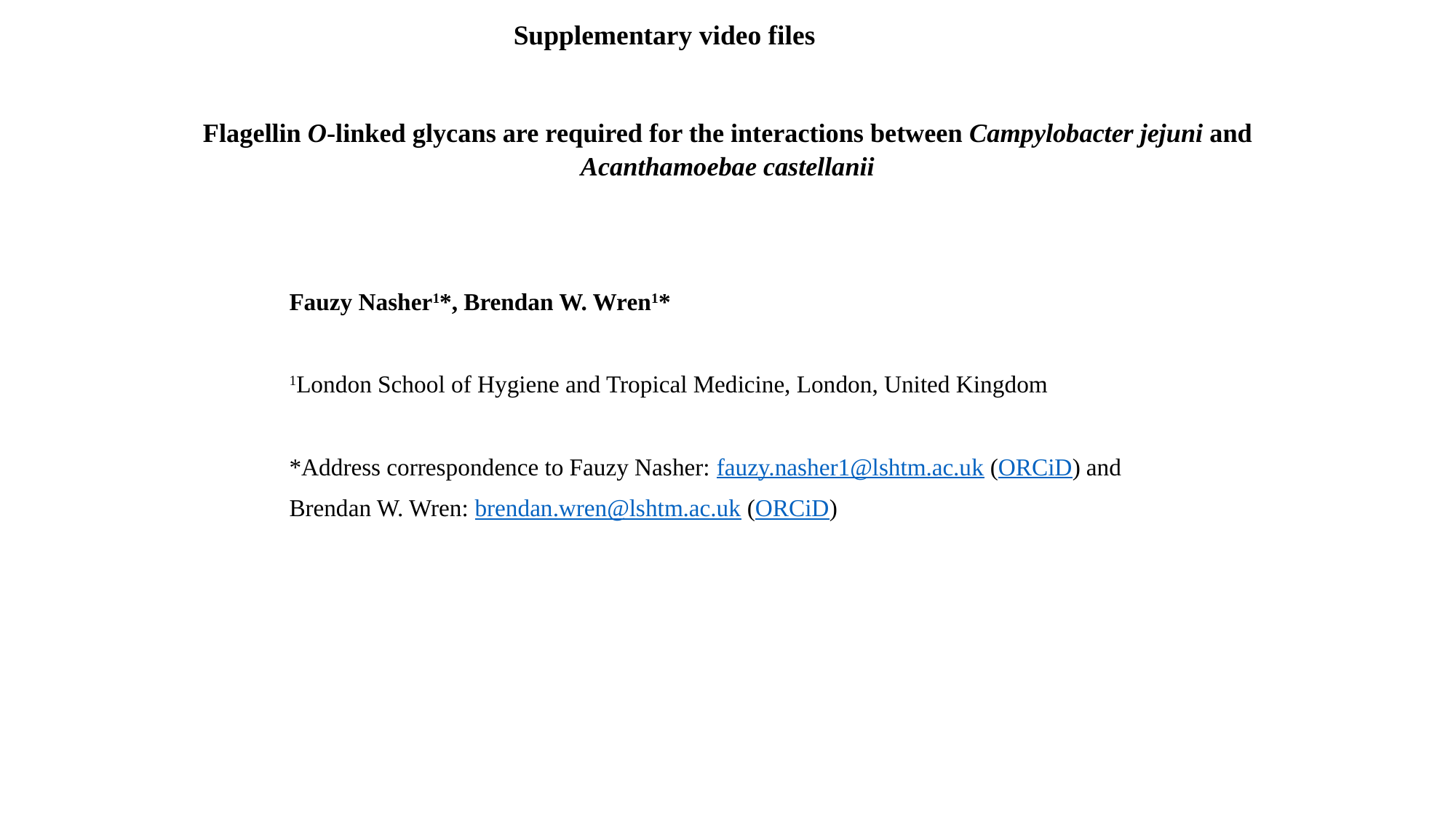

Supplementary video files
# Flagellin O-linked glycans are required for the interactions between Campylobacter jejuni and Acanthamoebae castellanii
Fauzy Nasher1*, Brendan W. Wren1*
1London School of Hygiene and Tropical Medicine, London, United Kingdom
*Address correspondence to Fauzy Nasher: fauzy.nasher1@lshtm.ac.uk (ORCiD) and
Brendan W. Wren: brendan.wren@lshtm.ac.uk (ORCiD)

## Slide 2
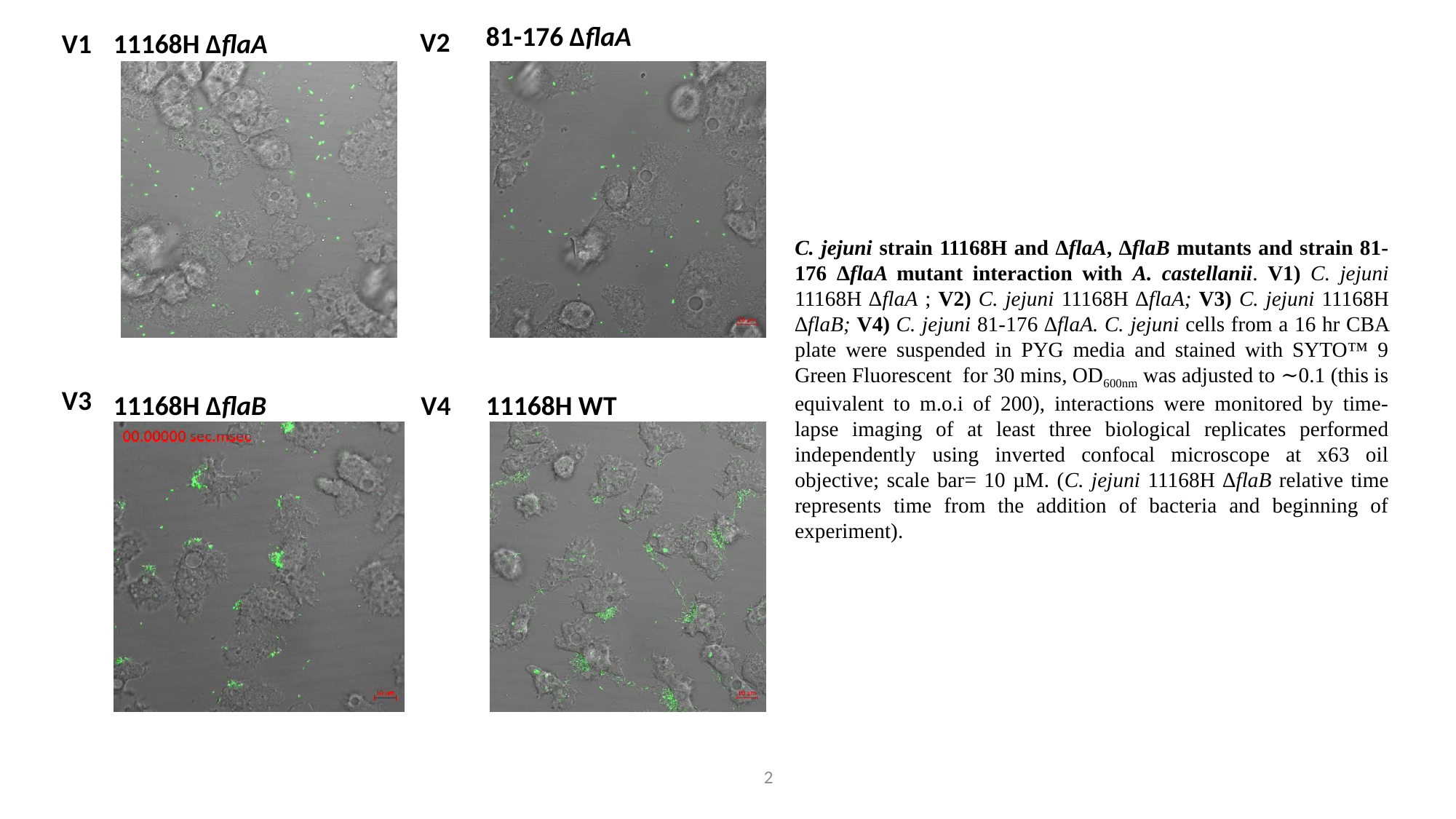

81-176 ∆flaA
V2
V1
11168H ∆flaA
C. jejuni strain 11168H and ∆flaA, ∆flaB mutants and strain 81-176 ∆flaA mutant interaction with A. castellanii. V1) C. jejuni 11168H ∆flaA ; V2) C. jejuni 11168H ∆flaA; V3) C. jejuni 11168H ∆flaB; V4) C. jejuni 81-176 ∆flaA. C. jejuni cells from a 16 hr CBA plate were suspended in PYG media and stained with SYTO™ 9 Green Fluorescent for 30 mins, OD600nm was adjusted to ∼0.1 (this is equivalent to m.o.i of 200), interactions were monitored by time-lapse imaging of at least three biological replicates performed independently using inverted confocal microscope at x63 oil objective; scale bar= 10 µM. (C. jejuni 11168H ∆flaB relative time represents time from the addition of bacteria and beginning of experiment).
V3
11168H ∆flaB
V4
11168H WT
2

## Slide 3
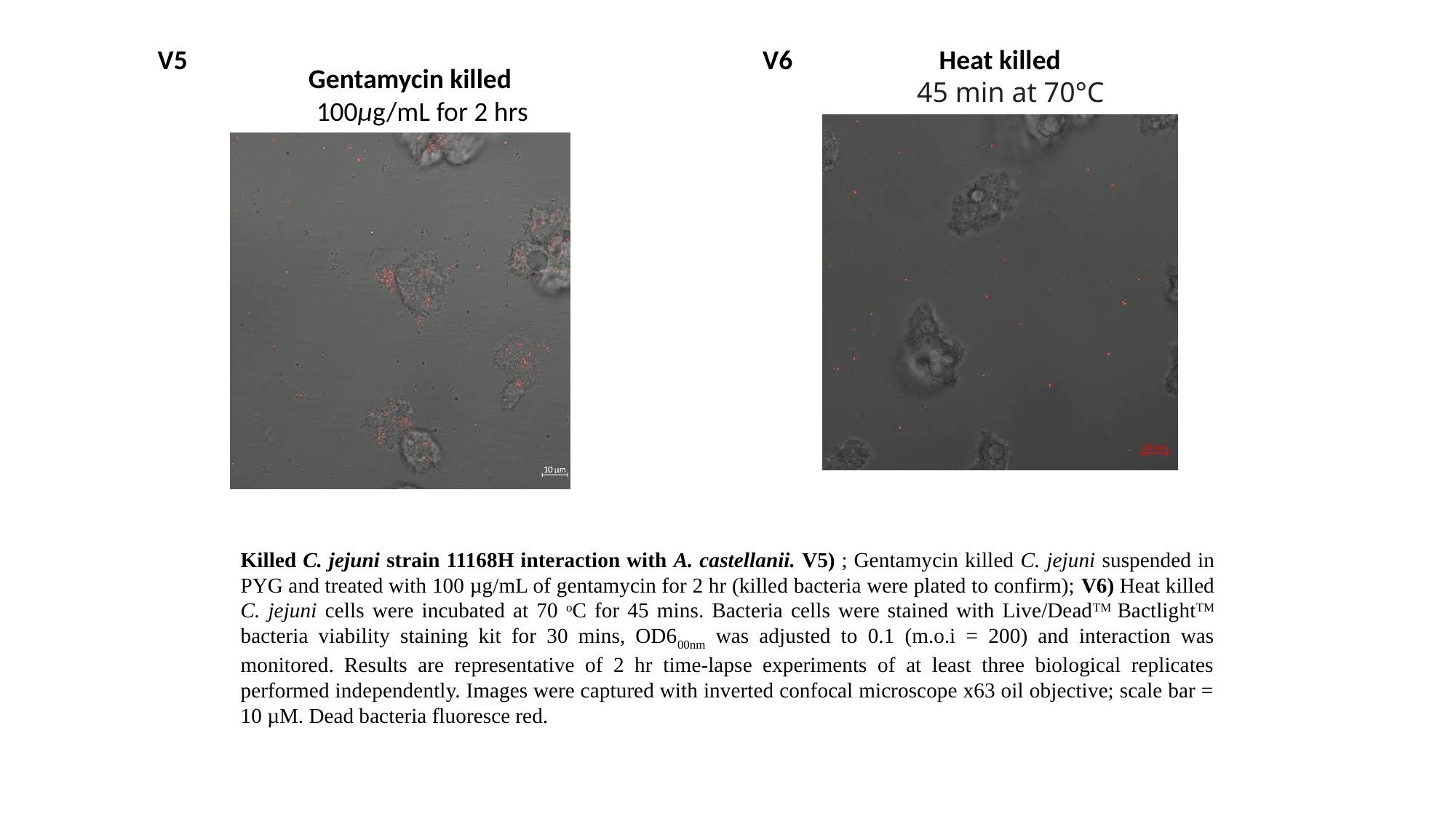

V5
V6
Heat killed
 45 min at 70°C
Gentamycin killed
 100µg/mL for 2 hrs
Killed C. jejuni strain 11168H interaction with A. castellanii. V5) ; Gentamycin killed C. jejuni suspended in PYG and treated with 100 µg/mL of gentamycin for 2 hr (killed bacteria were plated to confirm); V6) Heat killed C. jejuni cells were incubated at 70 oC for 45 mins. Bacteria cells were stained with Live/DeadTM BactlightTM bacteria viability staining kit for 30 mins, OD600nm was adjusted to 0.1 (m.o.i = 200) and interaction was monitored. Results are representative of 2 hr time-lapse experiments of at least three biological replicates performed independently. Images were captured with inverted confocal microscope x63 oil objective; scale bar = 10 µM. Dead bacteria fluoresce red.

## Slide 4
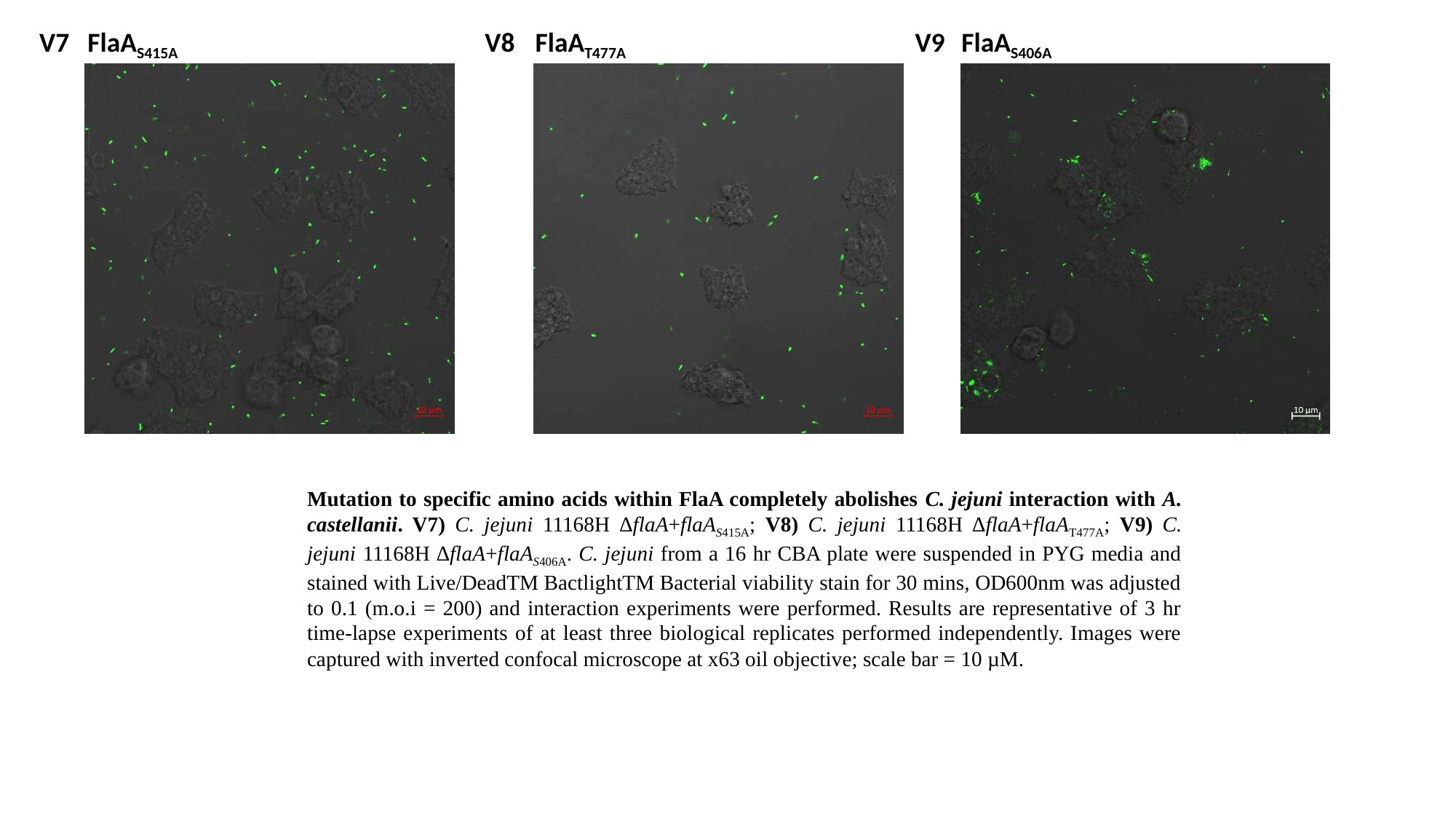

V7
FlaAS415A
V8
FlaAT477A
V9
FlaAS406A
Mutation to specific amino acids within FlaA completely abolishes C. jejuni interaction with A. castellanii. V7) C. jejuni 11168H ∆flaA+flaAS415A; V8) C. jejuni 11168H ∆flaA+flaAT477A; V9) C. jejuni 11168H ∆flaA+flaAS406A. C. jejuni from a 16 hr CBA plate were suspended in PYG media and stained with Live/DeadTM BactlightTM Bacterial viability stain for 30 mins, OD600nm was adjusted to 0.1 (m.o.i = 200) and interaction experiments were performed. Results are representative of 3 hr time-lapse experiments of at least three biological replicates performed independently. Images were captured with inverted confocal microscope at x63 oil objective; scale bar = 10 µM.

## Slide 5
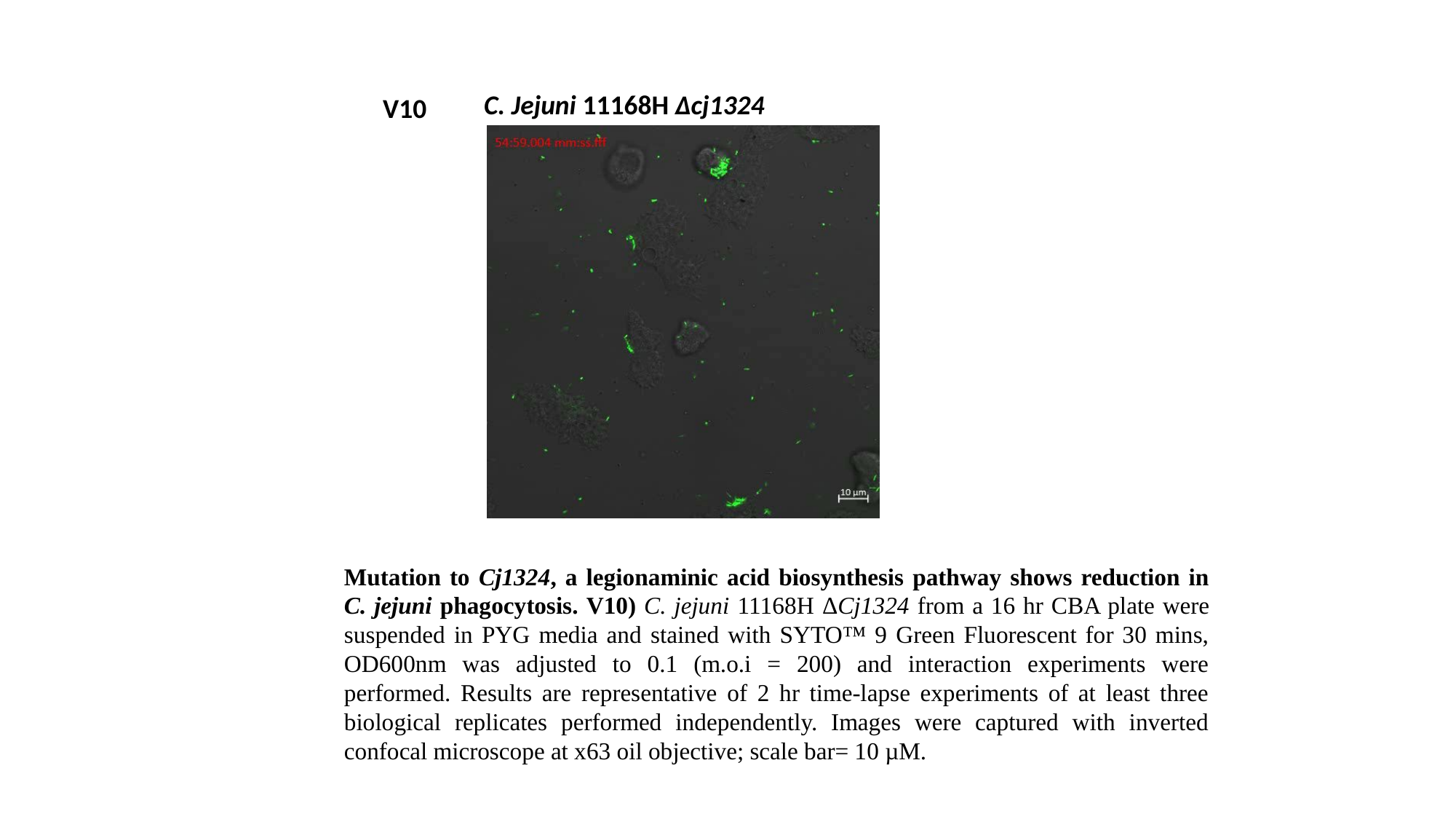

C. Jejuni 11168H Δcj1324
V10
Mutation to Cj1324, a legionaminic acid biosynthesis pathway shows reduction in C. jejuni phagocytosis. V10) C. jejuni 11168H ΔCj1324 from a 16 hr CBA plate were suspended in PYG media and stained with SYTO™ 9 Green Fluorescent for 30 mins, OD600nm was adjusted to 0.1 (m.o.i = 200) and interaction experiments were performed. Results are representative of 2 hr time-lapse experiments of at least three biological replicates performed independently. Images were captured with inverted confocal microscope at x63 oil objective; scale bar= 10 µM.

## Slide 6
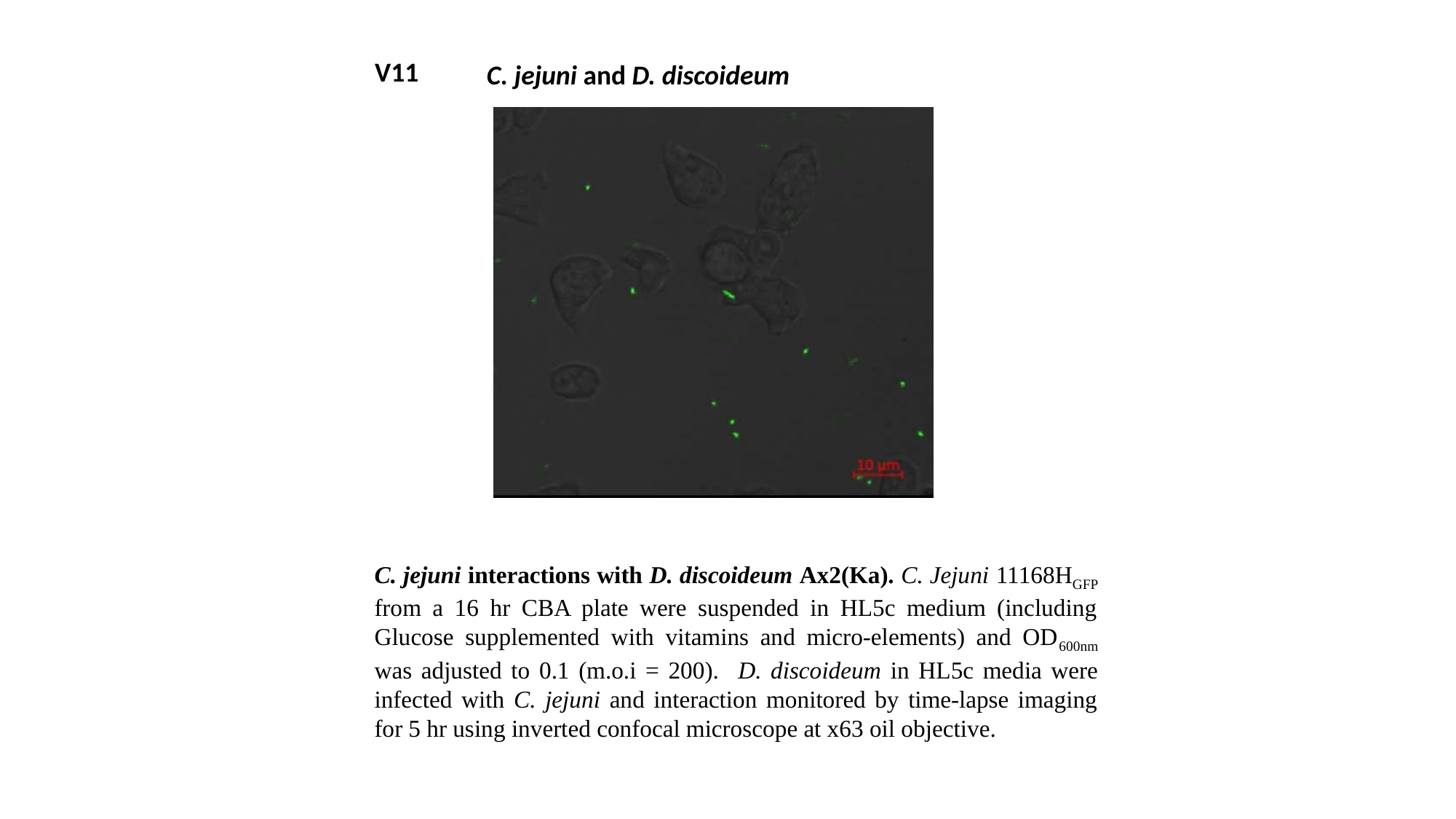

V11
C. jejuni and D. discoideum
C. jejuni interactions with D. discoideum Ax2(Ka). C. Jejuni 11168HGFP from a 16 hr CBA plate were suspended in HL5c medium (including Glucose supplemented with vitamins and micro-elements) and OD600nm was adjusted to 0.1 (m.o.i = 200). D. discoideum in HL5c media were infected with C. jejuni and interaction monitored by time-lapse imaging for 5 hr using inverted confocal microscope at x63 oil objective.
